# The use of click beetle pheromone traps to optimize the risk assessment of wireworm (Coleoptera: Elateridae) maize damage

**DOI:** 10.1038/s41598-020-64347-z

**Published:** 2020-05-29

**Authors:** Lorenzo Furlan, Barbara Contiero, Francesca Chiarini, Isadora Benvegnù, Miklós Tóth

**Affiliations:** 1 Veneto Agricoltura, Agricultural Research Department, 35020 Legnaro, PD Italy; 2 University of Padua, Department of Animal Medicine, Production and Health, 35020 Legnaro, PD Italy; 3 Freelance, 45011 Adria, RO Italy; 4 Plant Protection Institute, Centre for Agricultural Research, Budapest, POB 102, H-1525 Hungary

**Keywords:** Statistical methods, Databases, Environmental impact, Environmental economics, Sustainability, Entomology, Biosynthesis, Biochemical networks, Plant sciences, Health policy, Occupational health, Agroecology, Biodiversity, Ecological epidemiology, Ecological modelling, Population dynamics, Pollution remediation

## Abstract

Maize seeds are routinely coated with insecticide to target *Agriotes* spp. larvae (wireworms). However, in order to find fields where pest control is actually needed, it might be useful to estimate the adult *Agriotes* population levels and thus the pressure they exert, with a low-cost risk assessment tool, such as YATLORf (Yf) sex pheromone traps. A database containing 17 consecutive years (1998–2014) of field monitoring was analyzed, with information including both pheromone-trap catches of adults and maize-plant damage by wireworms. Significant associations were discovered between seasonal adult catches in-field, subsequent wireworm populations, and plant damage/yield reduction. When each trap contained over 1,100 *A. sordidus* adults and over 210 *A. brevis* adults one year prior (Y-1), the risk of 15%-plus plant damage in Year 0 (Y0) increased by 6 times and 37 times respectively when compared with lower numbers. More than 1,000 *A. brevis* adults/trap two years prior (Y-2) increased the risk of 15%-plus plant damage in Y0 by 13 times when compared with lower numbers. Cumulative thresholds were also found in Y-1 and Y-2 at the same site. Yf threshold values allowed us to detect fields with a negligible crop-damage risk and thus to reduce the use of insecticides.

## Introduction

Insecticides, including high-impact ones, such as neonicotinoids^1^, are used prophylactically worldwide in maize and other annual crops^2^. Most farmers apply soil insecticides at planting to combat wireworms (*Agriotes* spp. larvae), but they do not evaluate whether an economically damaging wireworm population is present^2^. This approach is used because farmers are generally unaware of Integrated Pest Management (IPM) strategies, little independent advice is available, and simple low-cost, low time-consuming monitoring tools are unknown to them^3^. Easy-to-use, economical, and reliable tools are needed to monitor the main arable crop pests if all farmers in EU Member States are to meet the compulsory objectives of Directive 128/2009/EC. Therefore, the availability of effective low-cost monitoring tools for Europe’s main soil pests (*Agriotes* spp.^4,5^) is a key factor for improving the situation and will make it easier to implement IPM in the main arable crops (maize, sunflower and canola). The use of these tools would allow researchers to locate areas and/or fields with pest populations below the economic threshold, as they account for the vast majority of the cultivated land^4,5^. One potential tool is the YATLORf trap (Yf)^6,7^. Produced by ROSA Micro S.r.l., this effective, non-saturable trap is baited with the pheromone lure of a given species, performs species-selective catches, and can be used to monitor adults of *Agriotes* species (click beetles) in a specific area. Yf traps have been developed and optimized for all major pest of *Agriotes* genus in Europe (*A. brevis* Candeze, *A. lineatus* L., *A. litigiosus* Rossi, *A. obscurus* L., *A. rufipalpis* Brullé, *A. sordidus* Illiger, *A. sputator* L., *A. ustulatus* Schäller)^6,8,9^, with some of these species being major pests in North America as well^10^. These traps may meet all of the requirements for arable-crop monitoring, partly because they are designed to monitor adults, the only stage that lives outside the soil. Furthermore, Yf traps have shown good potential for catching additional Elateridae species in other parts of the world (e.g. Iran and Western Asia^11^).

Since pheromone research began, attempts have been made to associate the magnitude of trap catches with population density or damage levels. One of the earliest examples of a full monitoring-scheme based on establishing a trap-capture threshold dates back to the 1980s and was performed on a tortricid pest (Lepidoptera) of green peas^12^. An early example of a quantitative monitoring scheme using beetle pheromones introduced a “Trap Index”^13,14^ to monitor the boll weevil *Anthonomus grandis* Boheman (Coleoptera, Curculionidae). In these and similar attempts, a very rough association at best was found between trap catches and subsequent damage. Where more precise relationships have been sought, they have proved to be inconsistent, either among localities, or from year to year. See the widely differing trap-catch thresholds for the codling moth *Cydia pomonella* L. (Lepidoptera, Tortricidae) established by various authors^15^.

In literature, only one study was found on the quantitative association between *Agriotes* catches in sex pheromone traps and wireworm populations^16^. Working in areas where *A. lineatus*, *A. obscurus* and *A. sputator* are the prevalent species, the authors concluded that they could find no relationship between *Agriotes* populations above ground (adults) and the populations below ground (wireworms), stressing that “for *Agriotes* click beetles, the proportion and distribution of adult male species trapped, at least with sex pheromone traps, may give a very misleading picture of the proportion and distribution of wireworm species in the soil”, a statement that contrasts strongly with the results of our research. However, the materials and methods described in the aforementioned study^16^ had major constraints, as fields were observed for one year only, methods for assessing wireworm density were not standardized, and key details, such as descriptions of trap management and methods for extracting larvae from soil cores, were missing. All of these points are essential if results are to be compared effectively with other papers on the same issue. A longer period of study using more consistent methods might have revealed significant associations between click beetles trapped in previous years and wireworm population levels at Y0, information that may have also provided a useful estimate of the actual damage risk for crops, especially by species such as *A. obscurus* and *A. sputator*, which move much less than *A. lineatus*^17^.

In the last few years, although a number of papers claim to have established significant associations between pheromone trap catches and crop damage, their final results seem to be weak and they are based mostly on data collected from just a couple of seasons^18–23^. The one exception is a recent paper^24^, who reported the results of a 16-year study conducted in the USA.

Our research assessed the thresholds for various *Agriotes* species to determine whether Yf traps could be used as an IPM tool. We believed that it would enable us to identify fields with a higher probability of hosting wireworm populations that could cause yield reduction and economic damage, with the aim of applying treatments only where necessary.

## Materials and Methods

This research used some of the maize fields from an extensive survey conducted in north-east Italy (area covered: 45.64 N, 12.96 E and 45.05 N, 11.88 E) from 1986 to 2014^4^. Pheromone-traps monitoring was included in this survey from 1998. The fields surveyed represent a balanced sample of agronomic conditions in north-east Italy. All the information, both entomological (collection of larvae, species determination) and agronomic (crop stand and damage, cultivation practices, yield), was directly collected during six inspections per field each year. All of the fields were untreated (no soil insecticide or insecticide-coated seeds), except for those seeded alternately with untreated and insecticide-treated maize in strips/plots, particularly where higher wireworm densities had been recorded with bait-traps^25^.

In the complete maize-field database used in this research, click beetles of at least one of the three main crop-threatening species in north-east Italy and southern Europe (*A. brevis*, *A. sordidus*, *A. ustulatus*) were monitored by using Yf traps.

**Figure 1:**
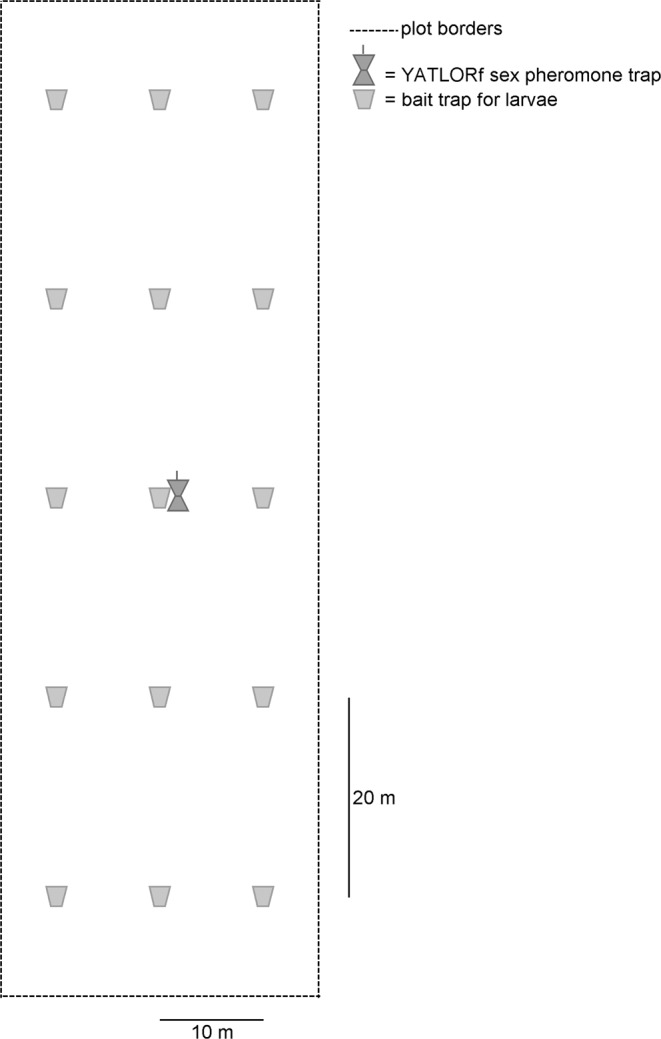
Experimental layout of Yf traps and bait traps for larvae in fields planted with maize.

The Yf traps were placed in the middle of the field one (Y-1) to two years (Y-2) before maize sowing and/or in the same year as maize cropping. Before maize cropping at Year 0 (Y0), bait traps for larvae were placed in the field as per Furlan^25^. When the field was planted with a narrow inter-row crop, such as winter wheat, a 1.5 m diameter circle of the plants around the Yf traps was destroyed. This was done in order to avoid interference with pheromone plume movements, which may have resulted in a reduction of click beetle catches. Afterwards, maize stand and soil pest damage were assessed with the methods described in a previous work^4^. The overall experimental layout is described in Fig. 1. The area monitored ranged from 0.2 to 1 ha, depending on the field size and shape.

### Assessment of *Agriotes* adult population

Yf traps produced by Italy’s ROSA Micro S.r.l. have been used^6^ for this research since the mid-to-late 1990s. They were baited with CSALOMON commercial pheromone lures made of polyethylene vials with lid (No. 730, Kartell S.p.A., Italy). The lures were produced by the Plant Protection Institute Centre for Agricultural Research, Budapest, Hungary. Their active ingredients were geranyl butanoate 15 *μ*l plus (E,E)-faarnesyl butanoate 15 *μ*l for *A. brevis*, geranyl hexanoate 30 *μ*l for *A. sordidus*, and (E,E)-farnesyl acetate 50 *μ*l for *A. ustulatus*, as described by Tóth *et al*.^9,26,27^. The dosages were also those described by Tóth *et al*.^9^. The traps were positioned at least 40 m from each other to prevent them interfering with one another, given that the range of trap attractiveness was about 30 m in a work by Sufyan *et al*.^28^. Similar results have been found using the same experimental method in Italy with the species *Agriotes brevis*, *Agriotes sordidus*, *Agriotes ustulatus* (unpublished data).

The trap’s white bottom was placed facing down, with its brown edge 1–2 cm beneath the soil. The baits were managed according to a standard seasonal schedule, in accordance with the life cycle and behavior of each species, as described below. On 10 March, the traps were baited with the sexual pheromone for *A. brevis* in the lower position and placed with their top side facing down. On 10 April, the captured insects were collected, and the trap was baited with the pheromone for *A. sordidus* in the middle position and placed with its top side facing down. On every inspection, the bottom was cleaned up, with soil and any possible residuals being removed. Around 10 May, the captured insects were removed and the pheromone bait for *A. sordidus* (at approx. 30 days) was swapped for a new one, again in the middle position, and the vial placed with its top side facing down. Around 10 June, the captured insects were collected and the bait for *A. brevis* in the bottom position was removed; the bait for *A. ustulatus* was put in the top position. Around 10 July, the captured insects were removed and the bait for *A. ustulatus* was swapped for a new one in the same position. On 10 August, the captured insects were collected, and the trap was removed.

Single traps were multi-baited after experiments demonstrated that the combination of lures used in this experiment had captured the same number of beetles as when the same traps were baited with single lures. An additional benefit of multi-baiting is that it lowers costs. Trap timing and lure placement were based on the species’ behavior: *A. ustulatus* click beetles swarm from early June to August^29^; and *A. sordidus* beetles swarm from April to August, peaking in May, thus much longer than *A. ustulatus*^30,31^. *A. brevis* behaves in a similar manner to *A. sordidus* (overwintering adults^32^), but has a longer swarming period, which starts slightly earlier than that of *A. sordidus*^32^. These pheromone traps catch both males and females, although males are prevalent^33–35^.

On every inspection, the insects were removed from the trap by following the procedure here summarized: firstly, the trap was removed from the soil; it was then placed inside a large plastic bag, which was opened so that the insects dropped inside; the bag was closed immediately after the trap had been removed. The trap was then returned to its initial position.

All the individuals were preserved in cool conditions (5–8 °C) for taxonomic identification^36^.

### Assessment of wireworm population

Bait traps made and used as per Chabert & Blot^37^ were deployed to assess wireworm population densities from late February until mid-April (for the method, see an aforementioned work^25^). The traps were deployed on a grid (20 m × 10 m apart, Fig. 1); a minimum of nine bait traps was placed per field and the sample area varied between 0.2 and 1 ha. The larger the area to be covered, the higher the number of traps placed. This research encompasses only fields monitored in spring.

### Agronomic practices

The following common agronomic practices were applied to all the fields: fertilization with 240–300 N kg, 70,000 to 80,000 seeds/ha, inter-row width 75 cm, pre-emergence plus post-emergence herbicide treatments determining very low weed densities. The following commercial hybrids were used: ANITA, COSTANZA, ALICIA, SENEGAL (1993–2001); TEVERE (2002–2004); DKC6530 (2005–2006); DKC6530, MITIC, KERMESS, KLAXON (2007–2008); DKC6666, NK FAMOSO, PR31A34, PR32G44 (2009–2010); DKC6677, PR32G44, NK FAMOSO (2011); KORIMBOS, KALIPSO and P1547 (2012–2014).

The vast majority of the fields were tilled conventionally, i.e. with ploughing, cultivator passages, harrowing and hoeing. A small number were tilled as minimum tillage.

### Maize plant damage assessment

When random untreated maize strips/plots (3 or 4.5 m wide) had been sown alternately with treated strips/plots, the most effective insecticides available were used^4^.

**Table 1:** Results of ROC analysis carried out on number of click beetles using the threshold of 15% for damaged plants.

Species and Monitoring year	Sample size	Positive group	AUC ± SE	95% CI	Cut-off	Sensitivity	Specificity	P
*A. brevis* Y0	275	5.07%	0.55 ± 0.09	0.49–0.61	>458	28.6%	98.8%	0.541
*A. sordidus* Y0	263	2.65%	0.60 ± 0.08	0.54–0.66	>445	85.71%	46.69%	0.23
*A. brevis* Y-1	248	5.24%	0.83 ± 0.07	0.77–0.87	>201	69.23%	98.3%	<0.001
*A. sordidus* Y-1	208	5.29%	0.74 ± 0.07	0.68–0.80	>1,110	54.55%	89.34%	<0.001
*A. brevis* Y-2	179	4.47%	0.70 ± 0.15	0.63–0.77	>458	62.5%	100%	0.168
*A. sordidus* Y-2	182	4.95%	0.67 ± 0.07	0.59–0.74	>409	88.89%	51.45%	0.019

Positive group = percentage of sample size for which the damage was >15%; AUC = area under the ROC curve (accuracy); SE = standard error; 95% CI = 95% Confidence Interval for AUC; Cut-off = criterion on the number of adults to predict a damage >15%.

**Table 2:** Association between seasonal click beetle catches of the field and plant damage in the following years.

Monitoring year	Species	Adult/trap per season	Plant damage	No. of records	Range of damage probability	Mean damage probability	RR (high vs. low)	SE	95% CI	Wald χ–square	P χ–square test
Year 0	*A. brevis*	≤450 (low)	≤15%	256	0.03–0.17	0.06	11.82	5.73	4.57–30.58	25.95	<0.001
			>15%	10							
		>450 (high)	≤15%	5	0.21–0.39	0.3					
			>15%	4							
	*A. sordidus*	≤400 (low)	≤15%	109	0.023–0.025	0.024	4.31	4.63	0.53–35.32	1.86	0.173
			>15%	1							
		>400 (high)	≤15%	147	0.025–0.046	0.028					
			>15%	6							
	*A. ustulatus* (empirical threshold)	≤1,000	≤15%	240	0.02–0.04	0.03	3.15	3.29	0.41–24.31	1.22	0.2703
			>15%	6							
		>1,000	≤15%	12	0.04–0.05	0.04					
			>15%	1							
Year −1	*A. brevis*	≤210 (low)	≤15%	230	0.02–0.12	0.03	37.61	20.09	13.20-107.15	46.1	<0.001
			>15%	4							
		>210 (high)	≤15%	5	0.15–0.99	0.48					
			>15%	9							
	*A. sordidus*	≤1,100 (low)	≤15%	169	0.02–0.08	0.04	6.14	3.53	2.0–18.98	9.94	0.002
			>15%	5							
		>1,100 (high)	≤15%	28	0.08–0.53	0.18					
			>15%	6							
	*A. ustulatus* (empirical threshold)	≤1,000 (low)	≤15%	186	0.022–0.029	0.024	3.39	3.67	0.41–28.35	1.27	0.259
			>15%	4							
		>1,000 (high)	≤15%	13	0.028–0.044	0.034					
			>15%	1							
Year −2	*A. brevis*	≤450 (low)	≤15%	169	0.01–0.22	0.03	40.95	25.39	12.14-38.10	35.83	<0.001
			>15%	3							
		>450 (high)	≤15%	2	0.37–0.68	0.52					
			>15%	5							
	*A. sordidus*	≤400 (low)	≤15%	83	0.043–0.047	0.046	6.85	7.2	0.87–53.71	3.36	0.067
			>15%	1							
		>400 (high)	≤15%	90	0.04–0.08	0.05					
			>15%	8							
	*A. ustulatus* (empirical threshold)	≤1,000 (low)	≤15%	191	0.02–0.03	0.02	12.93	13.73	1.61-103.71	5.81	0.016
			>15%	3							
		>1,000 (high)	≤15%	4	0.03–0.05	0.04					
			>15%	1							
Y-1 & Y-2*	*A. brevis*	other cases (low)	≤15%	74	0.02–0.33	0.05	25.67	—	8.46–77.83	38.94	<0.001
			>15%	3							
		>450 & >210 (high)	≤15%	5	—	0.84					
			>15%	0							
	*A. sordidus*	other cases (low)	≤15%	46	0.02–0.11	0.07	9.07	5.75	2.62–31.43	12.1	<0.001
			>15%	3							
		>400 & >1,100 (high)	≤15%	4	0.13–0.68	0.33					
			>15%	5							
	*A. ustulatus*	other cases (low)	≤15%	8	0.06–0.10	0.07	18.67	—	6.21–56.13	2.88	0.089
			>15%	2							
		>1,000 & >1,000 (high)	≤15%	—	0.12–0.14	0.13					
			>15%	1							
Year −1	*A. brevis* & *A. sordidus*	other cases	≤15%	180	0.01–0.11	0.04	22.14	10.34	8.85–55.34	43.92	<0.001
			>15%	6							
		both species exceeded the threshold	≤15%	2	0.11–0.68	0.27					
			>15%	5							
Year −2	*A. brevis* & *A. sordidus*	other cases	≤15%	166	0.02–0.07	0.04	46.94	28.2	14.47-152.35	41.07	<0.001
			>15%	3							
		both species exceeded the threshold	≤15%	1	0.07–0.18	0.11					
			>15%	5							

RR = Relative Risk; SE = Standard Error; CI = Confidence Interval; * thresholds considering years Y-1 and Y-2 at the same time.

One liter of the fungicide Celest XL (Metalaxil-m + Fludioxonil) per ton of seed was used to treat all the maize seeds planted. At the 2–3 and 6–8 leaf stages, 2 sub-plots of 20 m × 4 rows of maize per portion of untreated field (0.1–0.2 ha) or untreated strip (3–6 m × 100–300 m) were chosen at random and the plants observed. The location and the number of sub-plots were the same in both the untreated/treated strips and completely untreated fields. In order to assess wireworm damage on emerged plants, typical symptoms (e.g. wilting of central leaves, broken central leaf due to holes in the collar, wilting of whole small plants) were assessed and the soil around the unhealthy plants was dug up to a depth of 5–6 cm; any larvae found near the collar were collected and identified. Wherever maize plants were missing from the rows, the soil was dug up in order to assess possible wireworm damage to seeds and/or emerging seedlings. Total plant damage was calculated as the sum of damaged emerged plants, seedlings and seeds. In order to establish the effect of wireworm damage on yield, the same observations were made on the treated strips/plots, when present. Finally, the strips and the plots were harvested and the maize grain was weighted. Maize grain samples were collected and their moisture percentage measured with a Pfeuffer-Granomat (the same device was used for all samples each year). The four fields in which maize stands were irregular and plants had been damaged by factors other than wireworm activity (e.g. bird damage, low emergence due to low soil moisture, flooding) were not considered. In order to isolate the “wireworm damage effect”, analysis excluded the fields under considerable pressure from factors other than wireworms (e.g. other parasites such as viruses or rootworms, *Agrotis ipsilon* Hufnagel and *Diabrotica virgifera virgifera* LeConte). Fields in which the general conditions were good, but the soil insecticide had not worked properly and the stand of treated maize plots was not optimal were not used to evaluate the effect on yield (two cases only). Only damage assessments from untreated strips were registered in the database (the mean of the sub-plot assessments for each considered field). Larvae collected were identified with specific keys^38,39^.

### Statistical methods

All analyses were performed by SAS 9.3 (SAS Institute Inc., Cary, NC).

**Figure 2:**
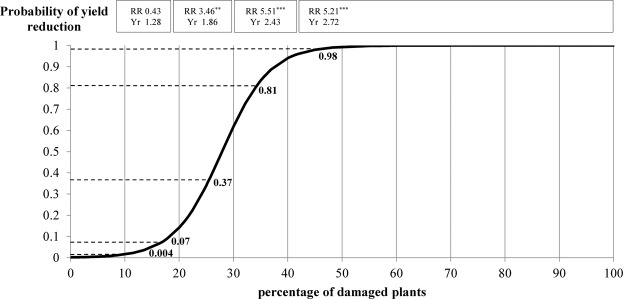
Relationship between the percentage of damaged plants and the probability of yield reduction (derived from a previous work^4^). RR = Relative Risk; *P < 0.05, **0.05 ≤ P < 0.01, ***0.01 ≤ P < 0.001; Yr = Average yield reduction (t/ha).

A 15% threshold of plants damaged by the wireworm population was used to predict accurately whether there was a significant risk of yield reduction (Fig. 2, derived from a previous work^4^). This threshold was calculated both mathematically, with a logistic regression model that considered the probability of yield loss based on the percentage of damaged plants, and empirically, with a 30-year-plus study showing that ≤15% plant damage never resulted in maize yield reduction^4^. With a 15% threshold, the probability of yield loss was less than 10%, whereas with a 40% threshold, the probability was about 90%.

A received operating characteristic (ROC) curve analysis was performed^40^ to establish accurate thresholds for the number of adults captured so that significant plant damage could be predicted. The number of adults/trap captured during the same year as maize sowing (Y0), previous year (Y-1) and two years before (Y-2) was analyzed according to the dummy variable based on 15% plant damage (recorded at Y0). The binary variable equaled 1 when plant damage was greater than 15%, which meant that there was a chance of yield loss^4^. The value of the dummy variable was 0 (zero) when plant damage was lower than or equal to 15%. The use of an ROC curve and binary variable established a risk threshold for plant damage (%), which enabled us to calculate a cut-off value, predictive of the same risk, for an associated continuous variable (number of adults/trap per season). The Youden criterion was used to set the optimum threshold for the number of adults^41^. This criterion maximizes both the sensitivity and specificity of a test. The area under the ROC curve (AUC) was taken as an index of accuracy. AUC values over 90% mean excellent accuracy, between 80% and 90% good, between 70% and 80% fair, and below 70% poor.

The adult populations were dichotomized as in the previous step, and the number of larvae was dichotomized as in a previous work^25^. Risk analysis was then performed on all data (n = 1,296 records, 4,867 hectares) by using a generalized linear model (GLM) with binomial distribution and a logit link function (PROC GENMOD). This approach was adopted to overcome the problem of the spatial auto-correlation and not-normal distribution of ecological data^42^.

The aim was to determine how each species (*A. brevis*, *A. sordidus*, *A. ustulatus*) each year could significantly increase the probability of more than 15% plant damage, and hence the probability of a yield loss in the present year (Y0). Combined thresholds for Y-1 and Y-2 were also used to assess the cumulative impact of a possible increase in adult population over the years. Relative risk (RR) and 95% confidence intervals (CI) were calculated to quantify the effect of click beetle population size on damage probability. The same approach was used to determine whether a link existed between the adult populations recorded in the previous years and the number of larvae recorded in the year the damage was caused (Y0) in order to estimate the risk that increased adult populations could increase the number of larvae.

On a sub-dataset (n = 218 records, 336 hectares) including the cases in which yield losses were recorded, further analysis was conducted to assess whether each species had caused a significant yield reduction. The number of click beetles and larvae recorded, dichotomized as in the previous steps (above and below the thresholds), and yield loss, classified as “significant reduction vs. not significant (n.s.) reduction”^4^, were entered into a 2 × 2 contingency table, with Fisher’s exact test being used to evaluate whether there was a link between the two variables.

## Results

### Yf catches vs. maize plant damage

#### ROC analysis

The ROC analysis detected a threshold of 458 for the number of *A. brevis* adults captured in the same year (Y0) plant damage was recorded, with method accuracy being 55% (P = n.s., Table 1). However, for data collected the years prior (Y-1), the threshold was 201 with 83% accuracy (P < 0.001). For data collected two years prior (Y-2), the threshold was 458, with 70% accuracy (P = n.s.). These results showed that, for this species, the number of adults most likely to cause damage was the number collected the year before the maize crop was sown. For *A. sordidus*, the results were the following: at Y0, n = 445 and accuracy = 60% (P = n.s.); at Y-1, n = 1,110 and accuracy = 74% (P < 0.001); at Y-2, n = 409 and accuracy = 67% (P = 0.019). For *A. ustulatus*, the ROC analysis failed, probably due to the presence of the other species hiding its effect. The harmful potential of this species is nevertheless generally low. An empirical threshold, based on extensive experience and plus decades of observations, was assumed in this case (n = 1,000).

The mathematical calculation of the thresholds produced values not observed in the fields in the experimental period and not rounded. To obtain values useful to an operative and practical approach, thresholds calculated with ROC analysis were rounded to the nearest ten, so that the result was no greater than the nearest observed value. The results were as follows: *A. brevis* were rounded down from 458 to 450 for Y0, up from 201 to 210 for Y-1, and down from 458 to 450 for Y-2. For *A. sordidus*: 445 was rounded down to 400, 1,110 to 1,100 and 409 to 400.

### Association evaluation

A significant association between seasonal click beetle catches in-field and plant damage was found at Y0 for *A. brevis* (Table 2) with an RR of 11.82 in cases of more than 450 adults/trap when compared with lower numbers. This means that when the number of adults/trap is >450 (the threshold calculated in the previous step) the risk of 15% plus plant damage, and thus a possible yield reduction, increases by about 12 times (Table 2).

The association between the number of adults captured at Y-1 and the damage recorded at Y0 significantly increased both for *A. brevis* and *A. sordidus*. The number of *A. brevis* adults captured at Y-1 was highly and significantly associated with maize plant damage the following year, with risk increasing by more than 37 times. For *A. sordidus*, when the number of captured adults per trap per year exceeded 1,100, the risk of 15%-plus plant damage rose by only 6.14 times. No significant association between Yf adult catches in Y-1 and plant damage at Y0 was found for *A. ustulatus*.

When we consider catches at Y-2, *A. brevis* and *A. ustulatus* were the species most significantly associated with damage at Y0. The RR was 40.9 when more than 450 *A. brevis* adults/trap per year were caught and 12.93 when more than 1,000 *A. ustulatus* adults/trap per year were caught compared with lower numbers. Remarkably, the number of *A. brevis* adults recorded each year (Y0, Y-1 and Y-2) was significantly associated with the damage recorded at Y0.

In almost all of the cases, when adult catches of *A. sordidus* and *A. brevis* exceeded their thresholds, there was an association with continuous soil cover with vegetation in the previous years (risk factor rotation C^4^). Therefore, when the number of adult catches exceeded the thresholds, there was a much higher risk than that based on agronomic risk factors alone. The damage risk of *A. ustulatus* appears, however, to be independent from any other risk factor and mainly associated with adult populations levels at Y-2.

### Combined thresholds

Yf catches were analyzed both for each *Agriotes* species for two consecutive years and for more than one *Agriotes* species at the same time.

In the former case, when records of click beetle catches were available for two consecutive years (Y-1, Y-2), a significant association was found between plant damage at Y0 and the number of both *A. brevis* and *A. sordidus* adults. The RR was 25.67 for *A. brevis* when more than 210 and 450 adults were caught per trap in Y-1 and Y-2 respectively, and 9.07 for *A. sordidus* when more than 1,100 and 400 adults were caught per trap in Y-1 and Y-2 respectively (Table 2). A risk ratio of 9.07 for the combined *A. sordidus* thresholds for two consecutive years exceeded the risk ratio for adults/trap found separately at Y-1 and Y-2, making the risk prediction more solid.

In the latter case, it was possible to establish the effects of more than one species exceeding their Yf seasonal thresholds for total catches at the same time. If, at year Y-1, both *A. brevis* and *A. sordidus* exceed their threshold, the probability of recording significant maize plant damage (>15%) was 22 times higher than any other combination (RR = 22.14). At year Y-2, the risk of plant damage being over 15% was nearly 47 times and statistically significant (Table 2).

### Yf catches vs. wireworm density

The 15% plant damage threshold, a cut-off for a serious risk of yield reduction, was also used to study the association between Yf catches and wireworm density assessed with bait traps. The density of every species, i.e. the number of larvae/trap recorded at Y0, was also significantly associated with the risk of plant damage. When the number of larvae/trap exceeded the wireworm thresholds established by long-term research^25^, the risk of observing 15%-plus plant damage rose by at least 21 times for *A. brevis*, 20 times for *A. sordidus*, and more than 15 times for *A. ustulatus* (Table 3).

**Table 3:** Association between the wireworm density (number of wireworms per trap) and plant damage at Y0.

Species	Larvae/ trap threshold	Damaged plants (N.)	No. of records	Range of damage probability	Mean damage probability	RR (high vs. low)	SE	95% CI	Wald *χ*–square	P *χ*–square test
*A. brevis*	≤1	≤15%	48	0.07–0.22	0.13	21	14.66	5.34–82.53	19.01	<0.001
		>15%	2							
	>1	≤15%	4	0.23–0.99	0.68					
		>15%	21							
*A. sordidus*	≤2	≤15%	238	0.02–0.21	0.04	20.05	7.61	9.53–42.20	62.38	<0.001
		>15%	8							
	>2	≤15%	8	0.22–0.99	0.58					
		>15%	15							
*A. ustulatus*	≤5	≤15%	91	0.01–0.05	0.02	15.33	17.26	1.69–139.23	5.88	0.0153
		>15%	1							
	>5	≤15%	15	0.05–0.46	0.13					
		>15%	3							

RR = Relative Risk; SE = Standard Error; CI = Confidence Interval.

In agreement with the results from Furlan^25^, the likelihood of wireworm populations exceeding the thresholds (*A. brevis* > 1 larva/bait trap, *A. sordidus* > 2 larvae/bait trap, *A. ustulatus* > 5 larvae/bait trap) also increased significantly where high click beetle populations had been found, the year prior for *A. brevis*, and two years prior for *A. ustulatus* (Table 4). Other cases were not considered due to the lack of numerosity in the database.

**Table 4:** Association between seasonal beetle catches of the field and the probability of the next establishment of wireworm populations.

Species and Monitoring year	Adults thresholds (No.)	Larvae thresholds (No.)	No. of records	Range of damage probability	Mean damage probability	RR (high vs. low)	SE	95% CI	Wald χ–square	P χ–square test
*A. brevis* Y-1	≤210 (low)	≤1	110	0.02–0.13	0.03	74	75.2	10.1–542.3	17.94	<0.001
		>1	1							
	>210 (high)	≤1	4	0.16–0.93	0.45					
		>1	8							
*A. sordidus* Y-1	≤1,100 (low)	≤2	83	0.025–0.028	0.027	Not est.				
		>2	3							
	>1,100 (high)	≤2	27	0.021–0.025	0.024					
		>2	0							
*A. ustulatus* Y-2*	≤1,000 (low)	≤5	60	0.01–0.05	0.02	20.33	26.12	1.64–252.17	5.5	0.019
		>5	1							
	>1,000 (high)	≤5	2	0.33–0.41	0.37					
		>5	1							

RR = Relative Risk; SE: standard error; CI = Confidence Interval; Not est. = not estimable; *empiric threshold for adults.

The risk ratio was 74 times higher for *A. brevis* and 20.33 times for *A. ustulatus* when the respective number of adults exceeded the thresholds. This means that when more than 210 *A. brevis* adults are caught per trap, the likelihood of its wireworm density being higher than 1 larva/trap increases by 74 times. When the number of *A. ustulatus* adults/trap is more than 1,000, the likelihood of its wireworm density being higher than 5 larvae/trap increases by 20 times.

### Yf catches vs. yield reduction

**Figure 3:**
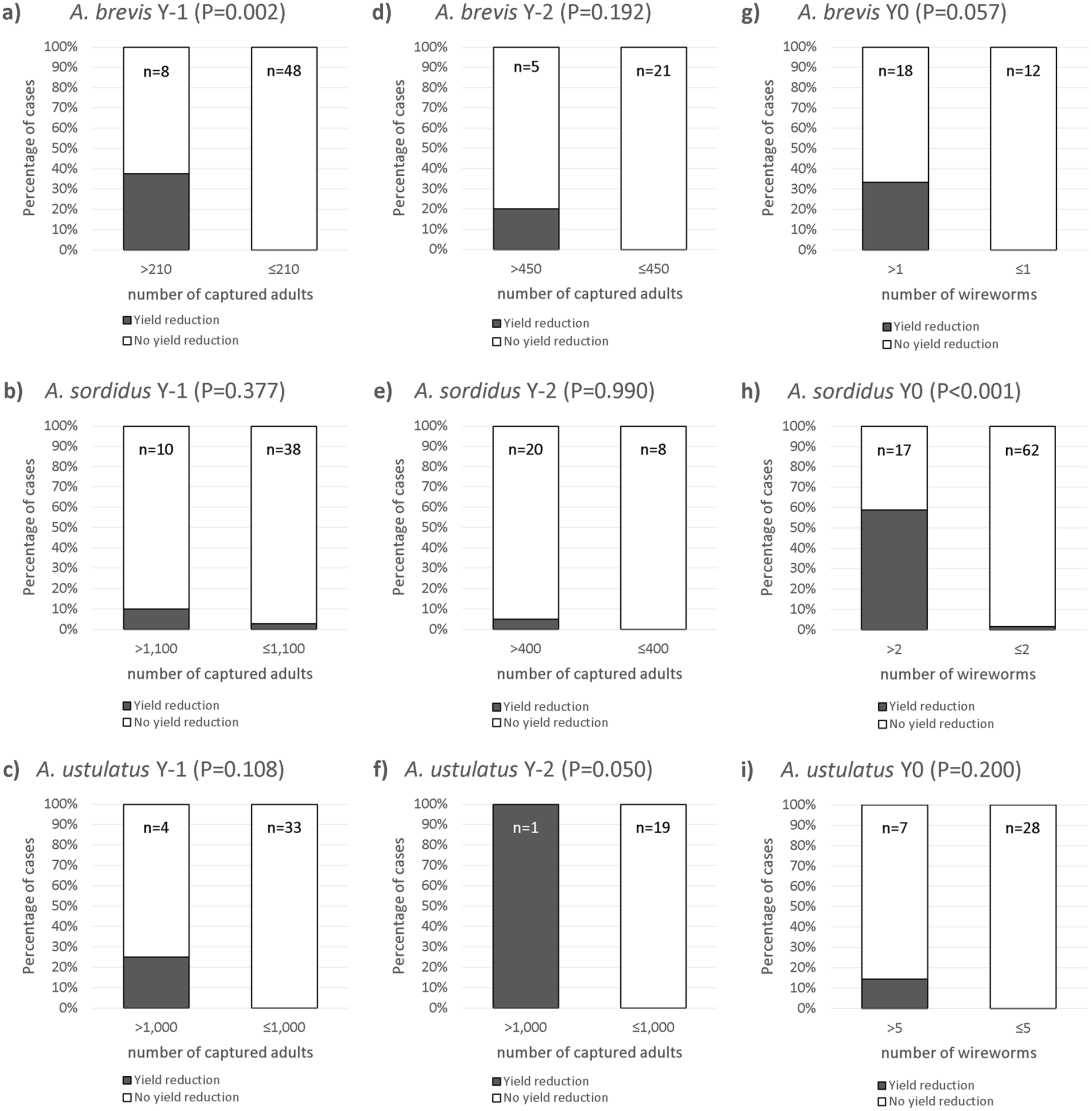
Percentages of cases of yield reduction (grey) vs. no yield reduction (white) for every click beetle and wireworm thresholds (P of Fisher exact test). **(a)** *A. brevis* adults Y-1; **(b)** *A. sordidus* adults Y-1; **(c)** *A. ustulatus* adults Y-1; **(d)** *A. brevis* adults Y-2; **(e)** *A. sordidus* adults Y-2; **(f)** *A. ustulatus* adults Y-2; **(g)** *A. brevis* wireworms Y0; **(h)** *A. sordidus* wireworms Y0; **(i)** *A. ustulatus* wireworms Y0. n = number of observed cases.

In a restricted dataset where yield production was recorded, it was possible to assess directly the association between seasonal catches of click beetles and the risk of yield reduction caused by wireworm damage. In Fig. 3, the trend for the three species is extremely clear. When the number of click beetles captured by Yf traps is higher than the thresholds, the probability of yield reduction tends to be higher. The likelihood of yield reduction is significantly higher for *A. brevis* (at Y-1; P = 0.002) and *A. ustulatus* (at Y-2; P = 0.05).

## Discussion

This is the first time that significant associations between seasonal click beetle catches in-field and subsequent wireworm density, as well as maize plant damage, have been found since pheromone traps were deployed to monitor click beetle species in cultivated fields. This discovery regards the three key species present in the area. A relationship between the *Agriotes* species prevalently captured by the pheromone traps and the main species found below ground as larvae had already been established by a previous study^43^, but this was not associated with the risk of maize damage at the time.

In our long-term study, when the number of *A. sordidus* adults/trap captured the previous year exceeded 1,100, the risk of breaking the 15% plant-damage threshold increased by 6 times when compared with values below this figure (Table 2). This is in perfect agreement with the biology of the species^30^. In fact, most of the *A. sordidus* larvae damaging a spring crop (e.g. maize) at the beginning of the growing season originate from eggs laid in the soil the previous spring (Y-1). A high number of click beetles found the previous year increases both egg-laying potential and the probability of having many larvae the next year if soil conditions are favorable.

Beetle-population monitoring the same year as maize planting has no practical implication. Nevertheless, it could be useful for mapping the potential risk of wireworm damage for an area, or in case a maize crop is scheduled to be sown again the following year. However, monitoring the beetle-population level two years prior to maize planting, could be useful in risk assessment, since part of the larvae does not pupate in the second year after egg laying, but grows for one more season. High beetle numbers combined with particularly favorable conditions in Y-2 could increase the number of larvae damaging the maize crop at Y0^30,31^.

As *A. brevis* has a very similar life cycle to *A. sordidus*^32,44^, a similar biological interpretation can be made for this species. As for *A. ustulatus*, high click beetle numbers (>1,000 adult/trap per season) in the summer of Y-2^29^ increased the risk of plant damage exceeding the 15% threshold by more than 12 times when compared with lower numbers (Table 2). This finding coincides with the life cycle of *A. ustulatus*, as larvae that can potentially damage maize in spring derive from eggs laid in the summer (late June – early August) of two years earlier^45^; the following spring, after egg laying, larvae are still too small to damage maize seeds and plants^45^. This means that when adult catches exceed the threshold, especially when associated with conditions favorable to the survival of young larvae in the summer of Y-2, the number of larvae potentially damaging the maize crop at Y0 can increase.

When adult monitoring is carried out in two consecutive years before maize seeding, results produce a threshold that estimates the combined values (Table 2), making risk prediction more reliable than one based on Yf seasonal catches in the cases of *A. sordidus* in Y-1 or Y-2 considered separately.

Since more than one *Agriotes* species can be present at the same site, a risk estimate considering more species simultaneously might be very useful. The combined adult threshold for *A. brevis* and *A. sordidus* gave risk factors of 22.14 and almost 47 times for Y-1 and Y-2 respectively, making risk prediction more reliable than one based on trap catches of a single species; in fact, the risk caused by more than 400 *A. sordidus* adult catches/trap was not statistically significant.

As the vast majority of cases with adult catches of *A. sordidus* and *A. brevis* exceeding the threshold were associated with continuous soil cover with vegetation in the previous years (risk factor rotation C^4^), combining these cases with the presence of agronomic risk factors makes risk prediction much more reliable. Data collected by using Yf traps from single fields might be further exploited by using them for a spatial estimate of click beetle populations with geostatistical methods, as demonstrated by a rural landscape research in northern Italy^46^.

While an aforementioned work^42^ studied how different factors can explain the presence or absence of *Agriotes* species, we estimated the probability of having high wireworm populations and a high damage risk for maize crop. Furthermore, the scale effect, i.e. the effect of the field generating auto-correlation effects, was low for the species studied in this work, as it was below 36% of the total deviation in models, that do not explain more than 43% of the total deviation.

Our work, which is based on many years of data, shows that there is a clear, statistically significant association between adult population levels and the presence of wireworms in the soil; it has also set a 15% plant damage cut-off that has led to fairly reliable adult and wireworm thresholds being established (Table 5) for the three main *Agriotes* species in southern Europe.

**Table 5:** IPM thresholds for Agriotes species in maize in southern Europe.

*Agriotes* species	Adults		Larvae
	Monitoring year	Click beetles/trap per season	Wireworms/trap at Y0
*Agriotes brevis*	Y-1	210	1
	Y-2	450	
*Agriotes sordidus*	Y-1	1,100	2
*Agriotes ustulatus*	Y-2	1,000	5

## Conclusion

This long-term research allowed us to assess the biological meaning of click beetle population levels, as assessed by Yf traps, for the main wireworm species in southern Europe. Yf adult catches can be used as a single risk factor for maize plant damage by wireworms (Table 5), or in association with other risk factors^4,5^. In the latter case, it is highly likely that this combination of factors will improve the reliability of wireworm damage risk assessment in maize crops. The thresholds established for the three southern Europe *Agriotes* species described herein might be the starting point for assessing the field thresholds of all the other *Agriotes* pests in Europe and North America. Based on the biology information available^29,31,32,45^, *A. brevis* adult thresholds may approximate the thresholds for the closely related species *A. obscurus* and *A. sputator*, while *A. sordidus* adult thresholds are likely to approximate the thresholds for *A. lineatus* and *A. rufipalpis*. The procedure and thresholds described herein can allow both farm-scale and area-wide monitoring, resulting in the drawing of risk maps in cultivated areas and enabling IPM of wireworms to be implemented at a low cost.

**Figure 4:**
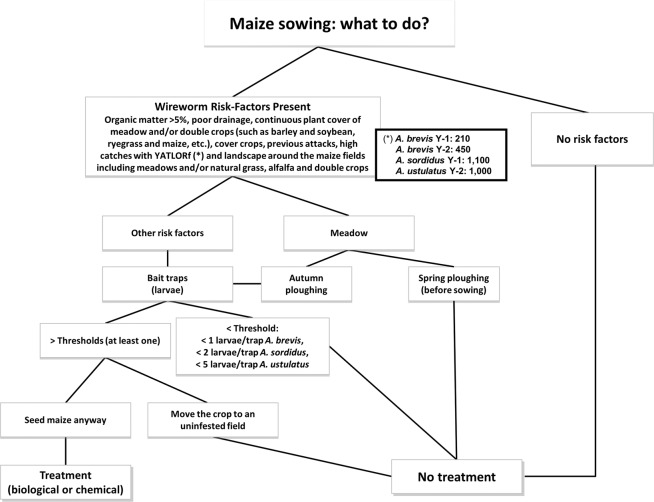
Integrated Pest Management (IPM) of wireworms in maize: steps to decide whether a treatment is needed (adapted from a previous work^5^).

These risk maps may be immediately useful for implementing IPM and for tackling the soil pests that threaten maize crops in many European regions and beyond. This may lead to a considerable reduction in the use of soil insecticides thanks to an improvement in the procedures described in a previous paper^5^, which can be updated with adult thresholds found in this research (Fig. 4). There will also be no negative repercussions on farmers’ income. The results of this work appear to provide one of the very first demonstrations of pheromone traps giving a reliable, consistent estimate of the risk of plant damage by crop pests based on observation made during several consecutive years.

Two additional innovative outputs of this research are the combined thresholds suggested:

- for two harmful *Agriotes* species exceeding their respective thresholds at the same time;
- for adult catches averaged from two subsequent years prior seeding (Y-1 and Y-2) and positively associated with maize plant damage at Y0.

This new information makes wireworm risk assessment much more reliable, especially when it is associated with agronomic risk factor evaluation, as it makes a significant improvement to the decision tree, which is the foundation of wireworm IPM (Fig. 4) and enables a dramatic reduction in soil insecticide use.

## Supplementary information

Supplementary information.
